# The epidemiology of infections with *Giardia* species and genotypes in well cared for dogs and cats in Germany

**DOI:** 10.1186/s13071-014-0615-2

**Published:** 2015-01-06

**Authors:** Louise Pallant, Dieter Barutzki, Roland Schaper, RC Andrew Thompson

**Affiliations:** School of Veterinary and Life Sciences, Murdoch University, Murdoch, WA 6150 Australia; Veterinary Laboratory Freiburg, P.O. Box 100120, 79120 Freiburg i. Br, Germany; Bayer Animal Health GmbH, 51368 Leverkusen, Germany

**Keywords:** *Giardia duodenalis*, *Giardia* spp, Domestic dogs, Cats, Germany, Molecular epidemiology

## Abstract

**Background:**

*Giardia* is now considered the most common enteric parasite in well cared for dogs and cats in developed countries. The ecology, epidemiology and clinical impact of infections with this parasite in such animals is still not fully understood due to variable results across different studies.

**Methods:**

Faecal samples were collected between 2009 and 2012 from privately owned cats and dogs in Germany presented to local veterinarians for a variety of reasons. *Giardia* positive samples were identified by microscopy and coproantigen methods. Total faecal DNA was extracted from *Giardia* positive samples and multilocus genotyping methods (18S rDNA, β-giardin, GDH) were applied. Relationships between host age, sex, and breed, season of presentation and the different species of *Giardia* detected were assessed.

**Results:**

A total of 60 cat and 130 dog samples were identified as *Giardia* positive. Potentially zoonotic *Giardia* was identified in both animal species. Cats had a similarly high rate of infection with the *G. duodenalis* and *G. cati.* Cats less than 1 year were more likely to have *G. duodenalis* than cats older than 1 year. Pure breed cats demonstrated a greater proportion of zoonotic species than mixed breed cats. In samples from dogs, *G. canis* (C and D genotypes) were identified most commonly. Male dogs were more likely to have *G. canis* (genotype D) than female dogs. The 18S rDNA PCR protocol was the most successful followed by the β-giardin and GDH (amplifying from 92%, 42% and 13% of samples respectively).

**Conclusions:**

The potentially zoonotic species *G. duodenalis* and *G. enterica* were found in cat and dog samples, with *G. duodenalis* found in greater numbers; however, this may be due to the detection techniques utilised. Cats appeared to show a relationship between *G. duodenalis* and *G. cati* with age and breed, which may be explained by different housing habitats for pure and mixed breed cats. The different success rates for the three loci utilised highlights the usefulness of the 18S locus as a screening tool, as well as the importance of using multiple loci for genotyping to fully determine the level of multiple infection of *Giardia* present.

## Background

The only way to achieve a meaningful understanding of the ecology and epidemiology of *Giardia* infections is to obtain more data on the distribution of species and genotypes in well-defined host populations. A variety of molecular genotyping tools are available which can be used in multilocus studies to produce such data. Interpretation is not always clear cut and is open to differing hypotheses but it is only with the accumulation of such data that a better picture of the ecology of *Giardia* infections will be obtained. This is particularly the case for *Giardia* in companion animals, dogs and cats. *Giardia* is now the most common enteric parasite in well cared for dogs and cats in developed countries [1-3]. This raises questions about the clinical significance of *Giardia* infections, *per se* and in cases of polyparasitism, and if this varies between different breeds of hosts [4,5].

Since dogs and cats are susceptible to different species of *Giardia* which vary in zoonotic potential, it is also important to obtain data on the frequency of infection in urban areas. Many surveys to date have focused purely on the assessment of *Giardia* prevalence in dogs and cats [2]. Some have attempted to determine the prevalence of specific species and their genotypes in various populations, often with an emphasis on determining their zoonotic potential [6]. While many studies frequently identify host adapted species as the most prevalent species within populations of dogs there are also contrasting studies where a higher prevalence of potentially zoonotic species have been identified [7-9]. Studies on cats describe a higher prevalence of potentially zoonotic species [2,10]. Many of these studies, however, rely on a single genetic locus for characterisation of the infections present in these companion animals. There is already detailed cautionary criticism of such an approach [6,11,12]. In brief, it is evident that the use of small fragments of highly conserved genetic targets (18S rDNA) can result in the misidentification of isolates, while preferential amplification of a species at a single genetic locus can mean that heterogeneous (mixed) templates are not identified. The present study was undertaken to provide additional data on the situation in Germany. Multiple genetic loci (18S rDNA, β*-*giardin and Glutamate Dehydrogenase (GDH)) were utilised not only with a view to identify the frequency of zoonotic species in dogs and cats but also to determine the frequency and impact of host adapted species on cohorts within this population of well cared for animals.

A revised taxonomy for the genus *Giardia* has been developed over the last few years [13] based on the original host specificity recognised by early taxonomists and reinforced by more recent genetic characterisation and molecular epidemiological studies. A summary of the proposed taxonomic revision for the genus is shown in Table 1. In the current study the revised species nomenclature is used; however, where discussion involves the subtypes within these species the sub-assemblage terminology is applied as described by Caccio *et al.* [14].

**Table 1:** **Species of** ***Giardia***

**Species**	**Assemblage**	**Host(s)**
*G. duodenalis*	A	Humans and other primates, dogs, cats, livestock, rodents and other wild animals.
*G. enterica*	B	Humans and other primates, dogs, cats, and some species of wild animals.
*G. canis*	C/D	Dogs and other canids
*G. bovis*	E	Cattle and other hoofed animals
*G. cati*	F	Cats
*G. simondi*	G	Rats

Adapted from Thompson and Monis [13].

## Methods

### Sampling strategy

**Figure 1:**
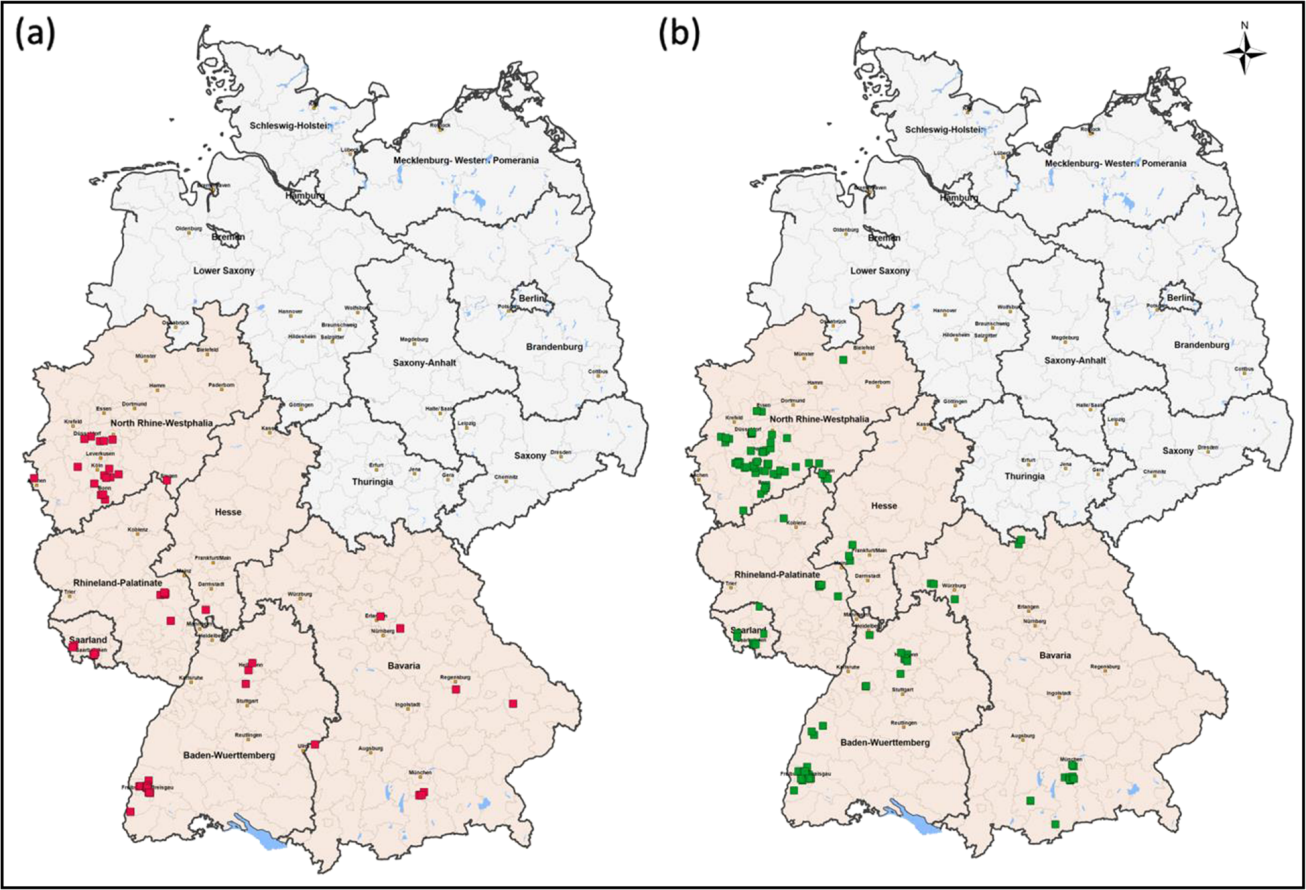
**Geographical origin of animals positive for** ***Giardia*** **spp. by SAF technique and copro-antigen test. (a)** *Giardia* positive cats (n = 60) and **(b)** *Giardia* positive dogs (n = 130).

Between October 2009 and January 2012 faecal samples from privately owned dogs and cats were routinely examined for endoparasites by the commercial Veterinary Laboratory Freiburg (Germany). The distribution of submitting veterinary clinics is illustrated in Figure 1(a-b). Animals were presented to veterinarians for a variety of reasons including gastrointestinal disorders, routine examination and vaccination or general health checks. Age, breed and sex data of *Giardia* positive cats and dogs provided the basis to analyse any relationships between these factors and the presence of species of *Giardia*. Samples detected as positive for *Giardia* spp*.* were collected from dogs between September 2009 and March 2011 with sampling occurring continuously throughout this period. Samples detected as *Giardia* spp*.* positive were collected from cats between October 2009 and February 2012. Due to low numbers of cyst positive cat samples the study was extended; therefore, collection occurred more sporadically throughout the sampling period than for the dog samples.

### Faecal examination

For detection of *Giardia* spp. samples were analysed by a coproantigen ELISA (ProSpecT® *Giardia* Microplate Assay, Remel Europe Ltd., distributed by Sekisui Virotech GmbH, Germany) as per manufacturer’s instructions or by sodium acetate formaldehyde SAF technique [15] to concentrate cysts of *Giardia*. Samples found positive for *Giardia* spp. by the identification of cysts and also by the coproantigen method were preserved in 70% ethanol for shipping.

### DNA extraction

Samples preserved in 70% ethanol were received at Murdoch University (Western Australia) in three consignments. In total, 60 cat samples and 130 dog samples were received for *Giardia* genotyping. Upon arrival at Murdoch sub-samples were transferred into 1.7 ml microfuge tubes and pelleted, the supernatant was removed and discarded. Total DNA was extracted from the pellets using the Maxwell® 16 Tissue DNA Purification Kit (Promega, Madison, USA) with the Maxwell® 16 SEV Instrument (Promega) according to manufacturer’s instructions. Total DNA extracts were stored at -20°C until processing.

### Amplification of 18S rDNA

Polymerase chain reactions (PCR) were carried out using 1 μl of both 1:4 diluted and neat total DNA template, 2.5 μl of 10 X reaction buffer, 2.5 μl of MgCl_2_ (25 mM), 0.1 μl T*th* Plus DNA polymerase (Fisher Biotech Perth, Australia), 1 μl of dNTPs (10 mM) (Promega), 1 μl of each primer (10 μM), 5% dimethyl sulfoxide (DMSO)(Sigma-Aldrich St. Louis, Missouri) and water-ultra pure grade, to a final volume of 25 μl. The first-round PCR conditions were: 96°C for 5 min for 1 cycle, 96°C for 45 s, 50°C for 30 s and 72°C for 45 s for 35 cycles followed by 72°C for 7 min, using RH11, 5′- CATCCGGTCGATCCTGCC −3′ and RH4, 5′- AGTCGAACCCTGATTCTCCGCCAGG −3′ from Hopkins *et al.* [16]. Template for the secondary PCR consisted of 1 μl of first round PCR reaction. Where a particularly strong or double banded product was produced a 1:4 dilution of the primary template that was used. Second-round PCR conditions were: 96°C for 5 min for 1 cycle, 96°C for 45 s, 55°C for 30 s and 72°C for 45 s for 35 cycles followed by 72°C for 7 min. For dog samples PCR primers GiarF, 5′- GACGCTCTCCCCAAGGAC −3′ and GiarR, 5′- CTGCGTCACGCTGCTCG −3′ [17] were utilised. For cat samples a semi nested approach was used using the primary oligo RH11 as the secondary forward primer and GiarR as the secondary reverse, allowing for a longer secondary fragment at the 5′ end of the sequence, where crucial nucleotide polymorphisms for *G. cati* are found.

### Amplification of β-giardin gene

PCR reactions used 1 μl of both the 1:4 diluted and neat DNA template, 2.5 μl of 10 X reaction buffer, 2.5 μl of MgCl_2_ (25 mM), 0.1 μl T*th* Plus DNA polymerase (Fisher Biotech Perth, Australia), 1 μl of dNTPs (5 mM) (Promega), 1 μl of each primer (10 μM), 5% DMSO (Sigma-Aldrich St. Louis, Missouri) and water ultra-pure grade (Fisher Biotech Perth, Australia), to a final volume of 25 μl. The first round PCR conditions were: 95°C for 5 min for 1 cycle, 95°C for 30 s, 50°C for 30 s and 72°C for 60 s for 40 cycles followed by 72°C for 7 min, using primers G7 5′-AAGCCCGACGACCTCACCCGCAGTGC −3′ and G759 5′-GAGGCCGCCCTGGATCTTCGAGACGAC −3′ [18]. Again 1 μl from the first-round PCR was used as template for the secondary PCR. Secondary PCR conditions were: 96°C for 5 min for 1 cycle, 96°C for 45 s, 55°C for 30 s and 72°C for 45 s for 35 cycles followed by 72°C for 7 min using primers 5′- GAACGAACGAGATCGAGGTCCG −3′ and 5′-CTCGACGAGCTTCGTGTT −3′ [19].

### Amplification of Glutamate Dehydrogenase gene (GDH)

PCR reactions used 1 μl of both the 1:4 diluted and neat DNA template, 2.5 μl of 10 X reaction buffer, 2.5 μl of MgCl_2_ (25 mM), 0.1 μl T*th* Plus DNA polymerase (Fisher Biotech Perth, Australia), 1 μl of dNTPs (5 mM) (Promega), 1 μl of each primer (10 μM), 5% DMSO (Sigma-Aldrich St. Louis, Missouri) and water ultra-pure grade (Fisher Biotech Perth, Australia), to a final volume of 25 μl. The primary PCR conditions were: 94°C for 5 min for 1 cycle, 94°C for 30 s, 50°C for 30 s and 72°C for 60 s for 40 cycles followed by 72°C for 7 min. One micro litre from the first round PCR reaction was used in the second-round PCR. Cycling conditions for second round PCR were: 94°C for 5 min for 1 cycle, 94°C for 30 s, 60°C for 30 s and 72°C for 60 s for 40 cycles followed by 72°C for 7 min. The primary PCR used primers GDHeF, 5′- TCAACGTYAAYCGYGGYTTCCGT -3′and GDHiR 5′-GTTRTCCTTGCACATCTCC -3′. The secondary PCR reaction used GDHiF 5′- CAGTACAACTCYGCTCTCGG -3′ and GDHiR [20].

### Sequencing

PCR products were purified using Agencourt® AMPure® XP PCR Purification (Beckman Coulter) in a 96 well format as per the manufacturer’s instructions. Sequence reactions were performed using the Big Dye Terminator Version 3.1 cycle sequencing kit (Applied Biosystems) according to manufacturer’s instructions. PCR products were sequenced with second round primers (1 μl [2.25 μM]). The cycling conditions for nucleotide sequencing were 1 cycle of 96°C for 2 min and 25 cycles at 96°C for 10 s, 50°C for 5 s and 60°C for 4 min. Reactions were electrophoresed on an ABI 3730 48 capillary machine. Sequence chromatograms were analysed using Sequencher® version 5.2 sequence analysis software (Gene Codes Corporation, Ann Arbour, MI USA (http://www.genecodes.com).

### Species, subtype and genotype identification

To confirm the species (assemblage) sequences were aligned with published sequences as described previously [21]. To determine *G. duodenalis* subassemblage and subtypes, sequences were aligned and compared with published sequences as defined by Feng and Xiao [6] (Table 2). It should be noted that the fragment of the GDH gene amplified by the protocol utilised here does not exhibit enough sequence divergence to distinguish between *G. duodenalis* subtypes A1 and A5 within subassemblage AI. In the results this ambiguity has been noted for isolates falling within this grouping at the GDH locus. For *G. enterica* (assemblage B) confident grouping was possible to species level only. Sequence comparisons were made using reference sequences for the alloenzyme based subassemblage groups BIII and BIV [14,22]. Where different sequences contained heterogenous bases or where different loci produced incongruent results samples were considered to contain templates from mixed species, subassemblages or subtypes.

**Table 2:** **Subassemblage subtype reference sequences**

**Species**		**Locus**	
G. duodenalis*		β-giardin	GDH
Subassemblage AI	Subtype		
	A1	X14185	AY178735
	A5	DQ984131	M84604
Subassemblage AII	A2	AY072723	AY178737
	A3	AY072724	EU278608
	A4		EF507657
Subassemblage AIII	A6	DQ650649	DQ100288
*G. enterica* ^#^			
Subassemblage BIII		AY072726	AF069059
Subassemblage BIV		AY072725	AY178738

Reference sequences as described by *Feng and Xiao [6] and ^#^Caccio *et al*. [14].

### Statistical analysis

Differences in infection ratios between groups (species, breeds, sexes) were tested by Chi-squared analysis. To test whether the prevalence of mixed infections differed from an independent random distribution pattern, observed frequencies were compared with those expected under a multiple kind lottery (MKL) model [23] using Chi-squared analysis. All statistical tests were performed with JMP® version 4.0.4 (SAS Institute Inc., Cary, NC, 1989–2007), at an alpha level of 0.05.

## Results

### Sample distribution

A total of 60 cat and 130 dog faecal samples were identified as *Giardia* spp. positive by both microscopy and coproantigen test and submitted to Murdoch University for genotyping. The geographical origins of these *Giardia* positive samples are illustrated in Figure 1(a-b). Samples from dogs originated from 53 different clinics with 1 sample as the lowest number submitted by any individual clinic and 8 as the highest. Samples from cats originated from 31 different clinics with 1 as the lowest number submitted by any individual clinic and 7 as the highest. Data for *Giardia* negative animals was not available for this study; the following results are therefore based on analysis of those samples that were submitted for genotyping only.

**Figure 2:**
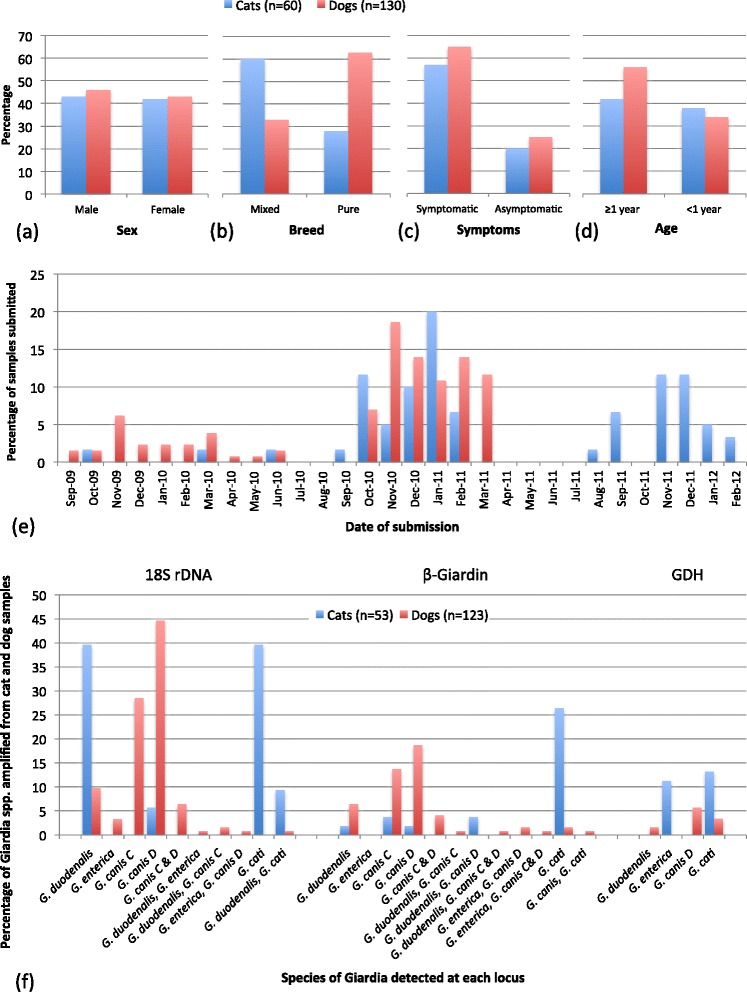
**Distribution of samples from cats and dogs positive for** ***Giardia*** **spp. by microscopy and coproantigen.** Percentage distribution is shown by: **(a)** sex, **(b)** Breed type, **(c)** Symptoms, **(d)** Age of animal, **(e)** Date of submission and **(f)** Species and mixed species combinations amplified at each locus used.

A summary of the distribution of *Giardia* positive samples collected for both animal cohorts by sex, breed, symptoms and age is illustrated (Figure 2(a–d). Symptom assignment (symptomatic or asymptomatic) was based on clinical history where animals were noted as displaying any gastrointestinal dysfunction on presentation to the veterinary clinic.

Samples from dogs, which were collected continuously throughout the study, demonstrated a marked fluctuation in season, with the submission of *Giardia* positive samples peaking in the autumn and winter months (Figure 2e). The cat samples were collected more sporadically throughout the sampling period; however, there was still a similar pattern identified with submission of *Giardia* positive samples also peaking in the autumn and winter months (Figure 2e). In addition to the seasonal peaks in *Giardia* there appeared to be relationships between the presence of *Giardia* cysts with age and with season in both cats and dogs. In the *Giardia* positive cats 62% of samples from younger animals were submitted in the autumn months while 65% of samples from older cats were submitted in the winter months (*X*^2^(3, N = 48) = 8.43, p = 0.04). In *Giardia* positive dogs overall no such trend was identified; however, in mixed breed dogs an opposite trend was identified with 61% of samples from younger animals submitted in the winter (*X*^2^(1, N = 39) = 3.13, p = 0.07). Significantly more polyparasitic infections (samples detected with concurrent infections of *Giardia* and one or more other parasites) were detected in dogs than in cats (*X*^2^ (1, N = 190) = 5.94, p = 0.02). In addition, *Giardia* positive dogs showed significant relationships between age and sex with polyparasitic infections, where younger animals (*X*^2^ (1, N = 117) = 5.5, p = 0.02) and female animals (*X*^2^ (1, N = 124) = 4.45, p = 0.04) bore a significantly greater number of mixed infections. This was the case for polyparasitism in general but particularly so for co-infections of *Giardia* spp. and *Cystoisospora* spp. in female dogs (*X*^2^ (1, N = 124) = 6.37, p = 0.01). In the cats there were too few samples to statistically test for risk factors of polyparasitism. Given the relationship between polyparasitism and sex in dogs, however, it is interesting to note that in the cats four out of the six samples identified with polyparasitic infections were in female cats. A summary of the polyparasitic infections identified in the cat and dog samples is presented (Table 3). Despite the low number of cat samples demonstrating polyparasitism with *Giardia* spp. a positive relationship between the presence of *G. duodenalis* (assemblage A) and co-infection with *Cystoisospora* spp. (*X*^2^(1, N = 45) = 3.36, p = 0.07) was also noted. There were no significant relationships between mixed infections of different *Giardia* spp. with any other factor.

**Table 3:** **Polyparasitic infections detected in dog and cat samples positive for** ***Giardia*** **spp**

**Polyparasitic infections detected with** ***Giardia*** **sp.**	**Cats**	**Dogs**
Hookworm*		1
*Toxascaris* leonina		2
*Toxocara canis*	1	8
*Angiostrongylus vasorum*		1
*Crenosoma vulpis*		2
*Cystoisospora* spp.	4	6
*Hammondia/Neospora* sp.		1
*Sarcocystis* spp.		2
*Toxoplasma gondii*	1	
*A. vasorum, C. vulpis, Trichuris vulpis*		1
*T. canis, Taenia* spp.		1
*T. canis, T. vulpis*.		1
*T. vulpis, Cystoisospora* spp.		1
Hookworm*, T. canis, Sarcocystis spp.		1
Hookworm*, *T. vulpis, Cystoisospora* spp.		1
*Capillaria* spp., *Cystoisospora* spp.		1
**Total**	**6**	**30**

*Ancylostoma or *Uncinaria.*

### Molecular genotyping

#### Cats

Of the 60 cat samples submitted for genotyping, 88.3% (53) were typeable at one or more loci with the 18S rDNA being the most successful and the GDH the least (Figure 2(f)). Overall, using one or more loci, single species of *Giardia* were identified in 67.9% (36/53) of cat samples, with multiple species identified in the remaining 32.1% (17/53). In cat samples that were amplified at multiple loci the genotypes concurred in 48% (12/25) of cases (including instances where a second locus confirmed the genotype identified at the first while simultaneously detecting another genotype – mixed template) (Table 4). In the remaining 52% (13/25) the genotype amplified at a second locus differed to the species detected at the first locus (Table 4).

**Table 4:** **Summary of molecular genotyping results for** ***Giardia*** **in dogs and cats**

**Host species**	**Sample size**	**Number amplified ≥1 loci**	**Number amplified ≥2 loci**	Assemblage concurrence at multiple loci*	**Assemblage divergence at multiple loci**
Cats	60	53 (88.3%)	25 (41.7%)	48% (n = 25)	52% (n = 25)
Dogs	130	123(94.6%)	59 (45.4%)	71% (n = 59)	29% (n = 59)

*Includes instances where a genotype identified at the first locus was again amplified at the second locus as part of a mixed sequence (indicating a mixed template). Figures are expressed as a percentage of the number of samples amplified at ≥2 loci.

Of the 53 samples that were successfully amplified, 18S rDNA PCR sequencing yielded genotype information for 94.3% (50/53), β-giardin PCR sequencing for 39.6% (21/53) and GDH PCR sequencing produced limited results with genotype information for only 24.5% (13/53) cat samples. The species and genotypes of *Giardia* amplified at each locus are summarised in Figure 2f. Sub-genotype information was obtained for 4 cat samples at the β-giardin and 6 at the GDH loci (Tables 5 and 6); however, due to the heterogeneity present at the GDH it was not possible to unequivocally assign a subtype to these isolates (Table 6).

**Table 5:** **Species and sub-genotypes for 53 cat and 123 dog samples at one, two or three loci**

**Host species**	**Single locus amplifications (n)**						**Multiple locus amplifications (n)**									
	**18S**		**GDH**		**β-giardin**		**18S**	**GDH**		**18S**	**β-giardin**		**18S**	**GDH**	**β-giardin**	
Cats	A	(13)	BIV	(2)	C	(1)	A,F	F	(1)	A	A1	(1)	F	BIV	F	(3)
	D	(3)					F	BIV	(1)	A	C	(1)	F	F	F	(3)
	F	(9)					F	F	(2)	A	D	(1)	A	F	F	(1)
										A	F	(4)				
										A,F	A2,D	(1)				
										A,F	A2,F	(1)				
										A,F	F	(2)				
										F	A1,D	(1)				
										F	F	(2)				
Dogs	A	(8)	F	(1)	C	(1)	D	D	(1)	A	A5	(1)	A	A1	A1	(1)
	A,B	(1)			D	(1)				A	BIII,D	(1)	A	A1	A5	(1)
	A,C	(1)			D	(1)				A,F	C	(1)	A,C	F	C	(1)
	B	(3)								B	C,D	(1)	D	D	A2	(1)
	C	(18)								C	A	(1)	D	D	C,D	(1)
	C,D	(5)								C	A1	(1)				
	D	(24)								C	BIII,D	(1)				
										C	C	(13)				
										C	D	(1)				
										C,D	A1,C	(1)				
										C,D	C,D	(2)				
										D	A5	(2)				
										D	C	(1)				
										D	C,D	(1)				
										D	D	(20)				
										D	A,C,D	(1)				
										D,B	BIII,C,D	(1)				
										D	D,F	(1)				
										D	F	(2)				

A = *G. duodenalis,* B = *G. enterica,* C and D = *G. canis*, F = *G. cati.* Heterogeneous sequences identified at a single locus are identified by letters separated by commas. The figure in parentheses indicates the number of samples with this result. For example in the first row 13 cats were detected with *G. duodenalis* only at the 18S, 2 cats were detected with *G. enterica* (BIV) only at the GDH, 1 cat was detected with *G. canis* (C genotype) only at the β-giardin, 1 cat was detected with both *G. duodenalis* and *G. cati* at the 18S as well as *G. cati* only at the GDH, 1 cat was detected with *G. duodenalis* at both the 18S and the GDH and finally 1 cat was detected with *G. cati* at both the 18S and the β-giardin while in the same sample *G. enterica* (BIV) was detected only at the GDH.

**Table 6:** **Sub-genotypes of** ***G. duodenalis*** **and** ***G. enterica*** **detected in cat and dog samples**

**Sub-genotypes detected**	**Cats**	**Dogs**
*G. duodenalis* (Assemblage A)		
subtype A1	2 (β-giardin)	3 (β-giardin)
subtype A2	2 (β-giardin)	1 (β-giardin)
subtype A5		4 (β-giardin)
subtype A1/A5		2 (2 GDH)
*G. enterica* (Assemblage B)		
subassemblage BIII		3 (β-giardin)
subassemblage BIV	6 (GDH)	

Overall, across all three loci the most commonly detected species in the 53 cat samples was *G. cati* 56.6% (30/53), followed by *G. duodenalis* 50.9% (27/53) and then low levels of *G. enterica* 11.3% (6/53), *G. canis* (genotype D) 11.3% (6/53) and *G. canis* (genotype C) 3.7% (2/53) (Table 5).

Mixed *Giardia* spp. included 10 samples with *G. duodenalis* and *G. cati* (one of these also included *G. canis* (D)), 4 samples included a mix of *G. enterica* with *G. cati* and 3 samples included a mix of *G. cati* with *G. canis* (C in one and D in two samples) (Table 5). There were no strong correlations between the presence of mixed species of *Giardia* with any other factor.

#### Dogs

Of the 130 dog samples submitted for genotyping, 94.6% (123) were typeable at one or more loci. As with the cat samples the 18S rDNA was the most successful and the GDH the least (Figure 2(f)). Overall, using one or more loci, single species or genotypes (including mixed *G. canis* C or D genotypes or mixed *G. duodenalis* subtypes) were identified in 75.6% (93/123) dog samples, with multiple species or genotypes identified in the remaining 24.3% (30/123). In dog samples that amplified at multiple loci the species or genotypes detected concurred in 71.2% (42/59) of cases (Table 4). In the remaining 28.8% (17/59) the species or genotypes amplified at a second locus differed to the genotype detected at the first (Table 4).

Of the 123 dog samples that were successfully amplified, 18S rDNA PCR sequencing was able to genotype 95.9% (118/123), the β-giardin PCR yielded genotype information for 48.7% (60/123) and the GDH PCR again produced limited results with genotype information for only 5.7% (7/123). Sub-genotype information was acquired for 11 dog samples at the β-giardin locus and 2 at the GDH locus. Again due to the heterogeneity present at the GDH it was not possible to unequivocally assign a subtype to these isolates (Tables 5 and 6).

Overall, across the three loci the most commonly detected species in the 123 dog samples was *G. canis* (D) 56.1% (69/123), followed by *G. canis* (C) 42.2% (52/123), and then lower levels of *G. duodenalis* 19.5% (24/123), *G. enterica* 6.5% (8/123) and *G. cati* 4.9% (6/123).

The presence of mixed *Giardia* spp. was detected in 15 dog samples, these included 13 samples with one or both of the zoonotic species (mainly *G. duodenalis*) with *G. canis* (genotype C or D or C and D) and 2 samples included a mix of *G. canis* (D or C and D) with *G. cati* (Table 5). There were no strong correlations between the presence of mixed *Giardia* spp. with any other factor although there was a trend for samples from female dogs to contain more mixed species (*X*^2^ (1, N = 117) = 3.28, p = 0.07). A mix of the two *G. canis* genotypes was detected in 15 (Table 5) dog samples.

#### Host adapted assemblages

Age and sex were identified as significant factors for the presence of the host adapted *G. cati* in samples from mixed breed cats only. Older and male cats in the mixed breed group had a significantly higher prevalence of this species of *Giardia* (*X*^2^ (1, N = 28) = 4.37, p = 0.04) and (*X*^2^ (1, N = 30) = 5.79, p = 0.02) respectively, than their younger or female counterparts. Due to the low number of samples it was not possible to carry out multivariate analyses on this data to determine a more complex relationship between the presence of *G. cati* with sex, age or breed in this group of animals.

Of the two recognised genotypes of *G. canis* (C and D) samples from male dogs had a significantly greater proportion of the D genotype detected in comparison to female dogs (*X*^2^ (1, N = 117) = 4.45, p = 0.04). This significance strengthened when only pure breed dogs were considered (*X*^2^ (1, N = 73) = 4.77, p = 0.03). An opposite trend was detected with the C genotype occurring in a greater proportion of samples from female pure breed dogs than from males (*X*^2^ (1, N = 72) = 3.56, p = 0.06). There was no similar correlation for the C genotype in the smaller cohort of mixed breed dogs. In addition, overall there appeared to be a negative association with the C genotype and symptoms (*X*^2^(1, N = 110) = 4.55, p = 0.03). Given the prevalence of *G. canis* C and D genotypes detected in the dog samples (40% and 53% respectively), under an MKL model the expected prevalence of a mixture of C and D genotypes in the dog samples would be 21%. The actual prevalence of such a mix was significantly lower at 11.5%, (*X*^2^ (1, N = 130) = 6.45, p = 0.01).

#### Zoonotic species

Due to the low number of *G. enterica* positive samples identified, the significance of zoonotic species was examined as a whole (both *G. duodenalis* and *G. enterica* together) or with *G. duodenalis* considered alone. A significantly greater proportion of the potentially zoonotic species were detected in the cats than in the dog samples (*X*^2^ (1, N = 174) = 21.87, p = <0.01). In both hosts zoonotic species of *Giardia* were as likely to be identified in samples with mixed genotypes as in those with only single genotypes detected. For the cat samples there was an association with the presence of potentially zoonotic *Giardia* spp. with age. Cats ≤1 year were significantly more likely than cats >1 year to harbour either of the two zoonotic species (*X*^2^ (1, N = 40) = 5.63, p = 0.02).

*G. enterica* alone was identified in too few samples to determine any significance with any particular factor in either of the animal groups. It is worth noting, however, that in both cats and dogs the greater proportion of samples identified with *G. enterica* came from animals ≤1 year (5/6 and 5/8 respectively). *G. enterica* was identified in 3 dogs all amplifying with the β-giardin PCR only. These sequences matched most closely to the BIII subassemblage grouping (Table 6). Conversely the 6 cats identified with *G. enterica* were identified with the GDH PCR only and all of these matched most closely to the BIV subassemblage grouping (Table 6). Nucleotide changes from subassemblage B sequences from both the β-giardin and GDH PCRs are presented in Table 7. Subtype information at multiple loci was obtained for 2 dog samples. Both grouped in subassemblage AI, one sample with subtypes A1 and A1/A5 and the second with A5 and A1/A5 at the β-giardin and GDH locus respectively.

**Table 7:** **Nucleotide changes in** ***G. enterica*** **subassemblage B isolates from cat and dog samples**

**Isolate**	**GDH**														
	**309**	**356**	**357**	**429**	**447**	**456**	**482**	**502**	**540**	**561**	**572**	**577**	**606**	**608**	**612**
STBIII reference sequence	C	T	T	T	T	G	T	G	C	C	T	G	C	T	G
STBIV reference sequence	**T**	.	.	**C**	**C**	.	.	.	**T**	**T**	.	.	.	.	**A**
1116074	**T**	**C/T**	**C**	**C**	**C**	**A**	**C/T**	.	**T**	**C/T**	.	.	**C/T**	.	**A/G**
1201420	**T**	.	**C**	**C**	**C**	**A**	.	.	**T**	.	.	.	.	.	**A**
1116246	.	.	**C**	**C**	**C/T**	**A/G**	.	.	**C/T**	.	**C/T**	.	.	**C/T**	**A**
1114218	**T**	.	**C**	**C**	**C**	**A**	.	**A**	**T**	.	.	.	.	.	.
1111290	**T**	.	**C**	**C**	**C**	**A**	.	.	**C/T**	.	.	.	.	.	.
1111426	**T**	.	**C**	**C**	**C/T**	**A/G**	.	.	**T**	**C/T**	.	**A/G**	**T**	.	**A/G**
	β-giardin														
	165	171	189	234	249	264	288	315	318	396	399	549			
STBIII reference sequence	G	C	A	G	C	G	C	C	C	C	C	C			
STBIV reference sequence	.	**T**	.	**A**	.	.	**T**	**T**	**T**	.	**T**	.			
1015139	**A**	**C/T**	.	**A**	.	**A/G**	.	**C/T**	**T**	**C/T**	.	**C/T**			
1102417	.	.	**A/G**	**A**	.	.	.	.	**T**	.	.	**C/T**			
1014340	.	.	.	**A**	**T**	.	.	.	**T**	.	.	.			

Nucleotide substitutions (in bold) are numbered from the ATG codon of each gene, dots indicate identity to the BIII reference sequence (GenBank Accession Nos.: GDH, AF069059; β-giardin AY072726).

In both cats and dogs there was a trend (significant in dogs) for *G. duodenalis* in particular to be identified more often in female animals, (*X*^2^ (1, N = 46) = 3.14, p = 0.08) and (*X*^2^ (1, N = 114) = 4.23, p = 0.04), in cats and dogs respectively. For the cat samples there was also an association with breed. There was a trend approaching significance in pure breed cats, which appeared to harbour a greater proportion of zoonotic species (*X*^2^ (1, N = 44) = 3.73, p = 0.05) in comparison to mixed breed cats. All *G. duodenalis* subassemblage sequences matched with 100% identity to their respective reference sequences.

#### PCR Amplification success of Giardia species in dogs and cats

The cat samples demonstrated a significantly higher PCR amplification failure rate at the 18S rDNA locus than dog samples 11/60 (18%) and 11/130 (8.5%) respectively (*X*^2^(1, N = 181) = 8.29, p = <0.01). There was a similar trend although not significant for the β-giardin PCR, with a failure rate of 39/60 (65%) and 69/130 (53%) for cat and dog samples respectively (*X*^2^(1, N = 183) = 3.10, p = 0.08). The reverse was true, however, for the GDH PCR where despite having a very high rate of failure in both species, the cat samples were more successfully amplified by this assay with 47/60 (78%) failing compared to 123/130 (95%) in the cats and dogs respectively (*X*^2^(1, N = 178) = 9.87, p = <0.01).

Mixed genotypes and/or species were detected in 49 samples in total (including samples with mixed *G. canis* C and D genotypes) and these were identified at all three loci to varying degrees (Table 5). As discussed previously, 14 of these samples were identified with heterogeneous sequences at a single locus, 10 (22%) at the 18S rDNA locus and 4 (7%) at the β-giardin locus. The remaining 35 samples were identified by the combined amplification at a second or third locus. Twenty five (51%) were identified with a combination of the 18S and the β-giardin PCRs and 2 (4%) by a combination of the 18S and GDH PCRs. The remaining 6 (12%) samples were identified by a combination of all three methods (Table 5).

**Figure 3:**
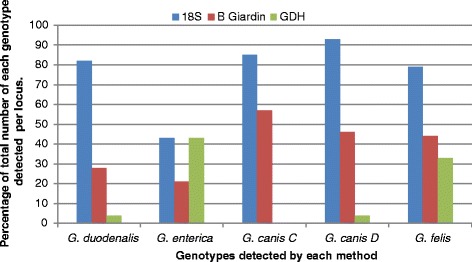
**The relative effectiveness of each PCR protocol utilised at amplifying each** ***Giardia*** **sp.** Effectiveness of each protocol is estimated by comparing the number of samples in which each protocol amplified a particular species compared to the total number of samples in which that species was amplified across all of the three protocols utilised.

The effectiveness of each PCR protocol to amplify each *Giardia* species was variable. A comparison of the number of times each PCR amplified each species as a percentage of the total species identifications (across all three PCR loci) is illustrated (Figure 3). Despite amplifying at least one species or genotype from 92% of all the samples submitted, the 18S rDNA PCR, performed comparatively poorly with *G. enterica* isolates amplifying from 6 of the 14 samples (43%) detected with this species. The second most successful protocol used, the β-giardin PCR (amplifying from 42% of samples overall), not only performed poorly when amplifying *G. enterica* (3/14, 14%) but in addition amplified from only 14 of a possible 50 *G. duodenalis* positive samples. The GDH protocol, despite being the least successful method utilised here, (amplifying only 13% of the total number of samples submitted) was comparable or better than the two other loci when amplifying *G. enterica*, equalling the 18S PCR in detecting 6 of 14 possible samples (42%) identified with this species. Interestingly, in both dogs and cats a trend showing that samples from males were more likely to amplify than samples from females was evident for the GDH PCR. When both animal species were combined this trend became significant (*X*^2^(1, N = 164) = 4.38, p = <0.04).

## Discussion

Previously *Giardia* has been identified as the most prevalent parasite found in both cats and dogs (12.6% and 18.6% respectively) in this geographical area [3]. This is one of the highest prevalence rates across several European countries [24]. Whilst in the current study data to determine the overall prevalence of *Giardia* was not collected, a stable prevalence of endoparasites in dogs and cats from the same area in Germany has been demonstrated for nearly 10 years [3,25].

The bias towards samples from symptomatic animals is probably a reflection of the sampling strategy of this study, in that animals were sampled once presented to a veterinarian rather than as part of a cross sectional survey. In addition, the variation between the two animal species with respect to breed (in cats more *Giardia* spp. positive samples were collected from mixed breed animals, while in dogs more were collected from pure breed animals) is likely to be a reflection of the distribution of these breed types within each of the two populations of well cared for cats and dogs. This is in contrast to one previous study which noted a reduced prevalence of *Giardia* spp. in mixed breed dogs [26]. There is substantial evidence supporting the increased susceptibility to *Giardia* spp. in younger dogs and cats [2,27] and this factor would account for the greater representation of dogs ≤1 year in this study although this was not demonstrated in the cat samples.

The overall seasonal distribution for both *Giardia* positive cats and dogs in this study closely corresponds to that of an earlier study in Germany [3]. Similar seasonal patterns have been identified in the USA and Argentina [2] with peaks in prevalence in the winter months, although this is not the case in other European based studies [28-30]. The reasons for such a persistent and striking seasonal fluctuation for *Giardia* spp*.* in well cared for dogs and cats in Germany, while neighbouring countries with a similar climate fail to show similar associations remain unclear.

The possibility of a relationship between younger cats and sample submission during autumn months is interesting, but this should be regarded with caution. *Giardia* positive cat samples were difficult to obtain and sampling for this cohort was not consistent throughout the study period. There did not appear to be a clear correlation between sample submissions in autumn or winter with the species of *Giardia* detected despite positive relationships between younger cats and the potentially zoonotic *G. duodenalis* and *G. enterica*, and similarly in older cats with the host adapted species *G. cati.* Such a relationship should be investigated with sufficient samples to enable multivariate analysis.

Previously, analysis of endoparasite infection in well cared for dogs and cats in Germany identified an increased prevalence of infection in animals less than one year of age [27]. This was also reflected here with a positive relationship observed between young dogs and polyparasitic infections with *Cystoisospora* spp*.* and *Toxocara canis* being the most common. Other studies have identified an increased risk in cats for *Giardia* spp. and co-infections with *Cryptosporidium* spp. and other coccidia [31]. While being too few to draw any statistical significance it should be noted that in the current study five out of the six co-infections in cats were with coccidian parasites. It is also interesting to note that coinfections with *Giardia* and *Tritrichomonas foetus* in cats [32,33] or *Pentatrichomonas hominis* in dogs [34] have also been identified, but examination for these parasites did not occur in this study.

In both animal groups presented here the most commonly detected species of *Giardia* was the host adapted species with which they are commonly associated, *G. cati* in cats and *G. canis* (C and D) in dogs. This agrees with many other studies [2,35-37] but is at odds with a previous study from the Munich area [7] and in the USA [8] where the authors found a predominance of zoonotic species of *Giardia*. The potentially zoonotic *G. duodenalis* and *G. enterica* were detected to varying degrees in both species of animal. Subtyping of some of these potentially zoonotic isolates identified several that are commonly associated with humans (Table 6) [6,10].

*G. enterica* was identified in too few samples to determine significance on its own; however, in both cats and dogs the greater proportion were identified in animals ≤1 year (5/6 and 5/8 respectively).

There was a significant difference between the two species of animals and the proportion of samples detected with potentially zoonotic species of *Giardia,* 50% and 21% in the cat and dogs samples respectively. The high prevalence of *G. duodenalis* in the cat samples in this study is consistent with other studies [10,38-40]. Discussion around the allocation of genetic subtypes into particular subassemblages and multilocus genotypes (MLGs) is confusing and at times contradictory or incomplete e.g. [10,14]. Without consistent well defined reference sequences, meaningful comparison and interpretation of zoonotic potential is difficult. For the purposes of this study *G. duodenalis* subtype reference sequences were used as defined by Feng and Xiao [6], although even here it appears that Portland-1 strain is used as a defining sequence for subtype A1 at the β-giardin locus and subtype A2 at the GDH. The subtypes detected in the present study suggest that the *G. duodenalis* isolates present fell within subassemblage AI. With the fragments we used, it was not possible to separate these further into MLG types. Likewise the *G. enterica* isolates present were split between BIII in the dog samples and BIV in the cats. The host distribution of *G. enterica* (assemblage B) is considered to be predominantly human, and, to a lesser extent, dogs and wildlife [10]; however, there are no obvious genotypes associated with zoonotic transmission.

There has been much discussion surrounding the pattern of transmission for zoonotic and host adapted species of *Giardia* particularly in dogs [4,41]. The hypothesis has been offered that transmission of host adapted species may be favoured by intensive contact between animals and these may then out-compete other non-host adapted species [42]. In the current study, the fact that cats had a much higher prevalence of non-host adapted species (or zoonotic species) of *Giardia* than the dogs may lend weight to this argument if this difference is placed in the context of the different animal behaviours. Owned dogs are generally gregarious animals that are often exercised in communal areas set aside particularly for that purpose, thus favouring the transmission of host-adapted *G. canis* [43]. Owned cats, however, are more territorial outside of home groupings [44] and when allowed to range freely do so in areas that have not been assigned for that purpose, potentially lessening their direct contact with other cats (in comparison with dog to dog contact). This behaviour therefore increases their propensity to acquire and retain the potentially zoonotic species of *Giardia.* In the present study, younger cats were seen to have a greater proportion of samples positive for the zoonotic species of *Giardia* in general. This was particularly so for the larger cohort of mixed breed animals. In addition, older animals in this mixed breed group were also more commonly infected with the host adapted *G. cati*. The opposite was true in the pure breed cats (although based on low numbers of samples) where older cats had a greater proportion of *G. duodenalis*. The difference in the pattern of infection between these two groups of cats (mixed and pure breed) may be related to housing conditions. Such restrictions may go some way in explaining the difference seen here. In both breed types in these well cared for animals one would expect younger animals to be kept indoors at least until vaccination was complete and the risk of them acquiring the zoonotic species would be increased due to their disproportionate contact with humans in comparison to other cats. As the animals mature they would be allowed free access to the outside and would therefore increase their contact with the environment contaminated with *G. cati* by other cats. If some of those animals, however, (more particularly pedigree animals) continue to be restricted to an exclusively indoor existence then they would have a significantly lower risk of contracting the host adapted *G. cati* and would therefore maintain their infections with the zoonotic species. In the current study, pure bred cats (particularly males) demonstrated a higher proportion of zoonotic *Giardia* spp. than their mixed breed counterparts (71% to 50% respectively). No data exists for the housing arrangements of the animals in this study or indeed for the proportions of pedigree cats that are housed exclusively indoors across European countries in general. There are studies from several countries such as the UK, USA, France, Canada and Australia that estimate varying levels of exclusively indoor housing (10% to 65%) of cats [45-52]. None of these studies give the breed status of the cats kept indoors although there is some evidence to suggest that expensive pedigree cats are more likely to be housed indoors [48]. In Australia, there is evidence supporting a greater level of exclusive indoor housing for pure breed cats. In Japan there has been some suggestion that this factor has an effect on the overall prevalence of *Giardia* spp. in such cats) [53,54]. It is therefore possible that pure breed cats may have been at greater risk of acquiring *G. duodenalis* and *G. enterica* due to greater proportions of them being restricted to an exclusively indoor environment. Further study is needed to investigate this association properly.

There are a number of studies that have examined the different species of *Giardia* affecting different populations of cats and dogs, e.g. shelter/stray and veterinary presentations, urban and rural, etc., and some have seen associations with *Giardia* presence and breed (Rottweilers) and sex (higher levels in female household dogs) [4,55]. As far as we are aware this is the only study to date that has attempted to correlate particular genotypes or species of *Giardia* to factors such as age, sex, breed or season within a single population type, i.e. well cared for cats and dogs. The possibility that female dogs and cats are more likely than their male counterparts to harbour not only potentially zoonotic *Giardia* spp. (rather than host adapted species) but also concurrent endoparasitic infections along with *Giardia* needs verification. Future studies should also aim to understand if this bias is due to behavioural (affiliative or gender based) or intrinsic biological reasons, if indeed this bias does exist. In addition, this is the first study to identify possible factors (age, sex and breed) involved in the acquisition of host adapted species or genotypes of *Giardia*. The relationship suggested in this study, between transmission of host adapted species/genotypes and their host’s sex, age or breed deserves further investigation. For clarity such studies would need to look at these factors within particular groups of animals, since previous studies comparing across differing groups (owned, stray or shelter) of animals have given variable results that are difficult to interpret in this context [41].

The observed difference between the actual and expected occurrence of mixed C and D genotypes in dog samples may well be explained by an association between these genotypes and gender as noted in this study. Further investigation is needed to rule out the role of any competitive exclusion that may be occurring between these two genotypes. Coupled with the observed association of C genotype and fewer symptoms as well as the genetic difference between these two genotypes [12] there may well be cause to revisit the classification of C and D genotypes within *G. canis*.

The success rates for each of the PCR methods used on the samples in this study are consistent with several other studies using large numbers of clinical samples [4,7,40]. There has been some criticism with regards to the inclusion of sequence data based on the 18S rDNA [12,41]. Indeed, while the limitations of the information gleaned from this locus (short, highly conserved fragments giving species (assemblage) level information only) are acknowledged here, had this locus been excluded from this study over half of the samples would have remained without any genetic characterisation whatsoever and many instances of mixed genotypes would have gone undetected. The results of this study clearly support the inclusion of the 18S rDNA locus in similar genotyping studies, particularly for difficult samples such as those from dogs; however, until a suite of reliable and well characterised loci are identified for *Giardia* genotyping it is critically important that multiple loci are utilised alongside, and that 18S rDNA data is not used in isolation. In this case the β-giardin was the next most cost effective option. Further work must include the development or enhancement of protocols including other locus options such as the triose-phosphate isomerase (tpi), GDH and possibly the more recently utilised ITS [56-59] to apply to such notoriously difficult sample types.

For the purposes of this study, the assumption has been made that each genotype detected within a sample represents a single infection from a non-recombining population and that where mixed genotypes were detected a coexisting multiple infection was present. Given this assumption, a total of 73 and 167 different isolates were identified in 53 cats and 123 dogs respectively. The use of multiple loci was responsible for detecting almost 70% of the mixed species/genotypes detected in this study, with the most successful combination being the 18S rDNA PCR with the β–giardin PCR. The use of multiple loci was attempted for all *Giardia* positive samples and while advocated by the authors here this ideal proved practically difficult and expensive to pursue. Such difficulties have been discussed previously [8,58] and the use of freshly extracted DNA has been recommended to improve the overall amplification success of the various loci included. This is not our experience; more recent studies have used freshly extracted DNA from fresh samples as a template for the same PCR protocols used here, resulting in the same variable success rates (Unpublished data). The relative strengths and weaknesses of each of the PCR protocols at amplifying from particular species of *Giardia* should be examined further and taken into account when deciding which methods to employ to detect potentially zoonotic species in animal samples. Preferential amplification of genotypes by particular protocols has been described [8]. In the present study the 18S rDNA PCR amplified fragments from 6 of the total 14 samples detected as positive for *G. enterica*. The β-giardin PCR amplified poorly from both zoonotic species, particularly *G. enterica* and while the GDH performed very poorly in general it did amplify from as many (and different) *G. enterica* positive samples as with the 18S rDNA protocol. This has been noted previously in studies from our laboratory [60]. There did appear to be a gender bias with the GDH protocol, however, this may be due to gender differences in the species or genotypes that are preferentially amplified. The likelihood is therefore, that for this study the number of potentially zoonotic species as well as the number of mixed genotypes actually present in these samples has been underestimated.

## Conclusions

This study has demonstrated the complexity of *Giardia* ecology in domestic dogs and cats, and has reinforced the influence exposure to different species of *Giardia* may have on which dominates under certain environmental and/or anthropogenic circumstances. As with other studies, the results reported here reinforce the public health significance of *Giardia* in companion animals with the occurrence of zoonotic species in dogs and cats, but demonstrate that it is impossible to extrapolate from one geographical area to another on the prevalence of zoonotic versus host adapted species, even in the same country. Polyparasitism, whether this involves mixed infections of *Giardia* species and/or other parasites, particularly *Cystoisospora*, is clearly an aspect of enteric parasitism that requires further study, especially in dogs and cats less than one year of age. Is *G. duodenalis* or *G. canis*/*G. cati* more pathogenic in dogs and cats, and what is the impact of co-infections with *Cystoisospora* in young animals particularly prior to weaning? Finally, our results have demonstrated the importance of taking a multilocus approach in studies on the molecular epidemiology of *Giardia* infections, and particularly the relevance of including 18S rDNA as one of the loci examined.
